# Biosensing Technologies for *Mycobacterium tuberculosis* Detection: Status and New Developments

**DOI:** 10.1155/2011/193963

**Published:** 2011-03-16

**Authors:** Lixia Zhou, Xiaoxiao He, Dinggeng He, Kemin Wang, Dilan Qin

**Affiliations:** State Key Laboratory of Chemo/Biosensing and Chemometrics, College of Biology and College of Chemistry and Chemical Engineering, Hunan University and Key Laboratory for Bio-Nanotechnology and Molecule Engineering of Hunan Province, Changsha 410082, China

## Abstract

Biosensing technologies promise to improve *Mycobacterium tuberculosis* (*M. tuberculosis*) detection and management in clinical diagnosis, food analysis, bioprocess, and environmental monitoring. A variety of portable, rapid, and sensitive biosensors with immediate “on-the-spot” interpretation have been developed for *M. tuberculosis* detection based on different biological elements recognition systems and basic signal transducer principles. Here, we present a synopsis of current developments of biosensing technologies for *M. tuberculosis* detection, which are classified on the basis of basic signal transducer principles, including piezoelectric quartz crystal biosensors, electrochemical biosensors, and magnetoelastic biosensors. Special attention is paid to the methods for improving the framework and analytical parameters of the biosensors, including sensitivity and analysis time as well as automation of analysis procedures. Challenges and perspectives of biosensing technologies development for *M. tuberculosis* detection are also discussed in the final part of this paper.

## 1. Introduction

*Mycobacterium tuberculosis* (*M. tuberculosis*) is a much dangerous pathogenic bacterium that causes tuberculosis (TB)—one of the leading causes of death from infectious diseases [1]. Currently, about one-third of the human population is infected with TB worldwide [2]. The infection of TB is a serious public health concern because it may emerge as a complication of acquired immune deficiency syndrome infection. The rapid diagnosis and treatment of infectors is considered crucial for the effective control of TB because one patient is known to transmit the disease to 12–15 people/year on average through respiratory tract infection [3]. Areas including clinical diagnosis, water and environmental analysis, food safety, and biodefense are quite critical for sensitive detection of *M. tuberculosis*. Therefore, it is very important and crucial for global public health protection to detect, identify, and quantify *M. tuberculosis*. Traditional microbial culture-based tests are the most common methodologies currently used [4, 5]. Usually these methods involve cell culture, cell counts, and cell enrichment, but this process is time consuming and laborious, especially for the slow-growing bacteria like *M. tuberculosis.* The heavy global public health burden of TB worldwide demands for the development of more rapid and sensitive detection methods. To date, many methods and techniques have been developed for rapid detection of *M. tuberculosis*, such as polymerase chain reaction (PCR) [6–9], latex agglutination [10], enzyme-linked immunosorbent assay (ELISA) [11–13], radiometric detection [14], gen-probe amplified *M. Tuberculosis* direct test (AMTDT) [15], TB rapid cultivation detection technique, such as MB/Bact system, BactecMGIT 960 system [16, 17] and flow cytometry [18]. These methods are more sensitive and rapid than the traditional microbial culture-based methods, as summarized in Table 1. However, they cannot provide the detection results in real-time and most of these methods are centralized in large stationary laboratories because complex instrumentation and highly qualified technical staff are required. As a result, the development of portable, real-time, sensitive, rapid, and accurate methods for* M. tuberculosis *detection is essential to effectively prevent TB infection [19, 20]. 

In recent years, with the improvement of sensing technology research, biosensing technologies are well suited for the purpose. The areas where biosensors show particular importance are clinical assay, disease diagnostics, food security, bioprocess, and environmental monitoring. The importance of biosensors results from their high specificity and sensitivity, which allows the detection of a broad spectrum of analytes in complex sample matrices (saliva, serum, and urine) with minimum samples pretreatment [21–32]. This short review mainly focuses on the discussion of piezoelectric quartz crystal biosensors, electrochemical biosensors, and magnetoelastic biosensors as examples to summarize the development of different biosensors for the use for *M. tuberculosis* detection. Lastly we discuss the future perspectives of biosensors for bacteria, viruses, and other microorganisms' detection.

## 2. Development of Biosensors for *M. tuberculosis* Detection

The biosensor for *M. tuberculosis* detection is generally defined as a compact analytical device incorporating a biological sensing element with a physicochemical transducer. Depending on the biological element employed, nucleic acid and antibody-based biosensors have been developed. According to the methods of signal transduction, it can be divided into piezoelectric, electrochemical, and optical biosensors for *M. tuberculosis* detection [33–35]. By comparison with culturing system detection and nucleic acid amplification systems detection, there are many advantages associated with the use of biosensing technologies as a rapid and sensitive detection method for* M. tuberculosis* detection, which allow the detection of *M. tuberculosis* in complex sample matrices (serum, urine, or saliva) [36]. For example, (1) it is high specificity by using the biological sensing elements, which can distinguish the targets from other microorganisms; (2) the response time is rapid; (3) it has the capability to provide continuous data with minimal quantity of the samples; (4) it can detect the analytes on-line due to the sensor and the signal transducer in series; (5) some of the biological elements, which are used in detection process, can be reused for other samples [37]. 

### 2.1. Piezoelectric Quartz Crystal Biosensors

The piezoelectric quartz crystal (PQC) sensor is one of the new bioelectrochemical devices used for direct detection of* M. tuberculosis*. By combining the high sensitivity with mass and surface characteristics of quartz crystal, such as viscosity, density, dielectric constant, and conductance with the high specificity of biological molecules, the PQC sensor has attracted many analysts because of its high sensitivity, low cost, small size, online detection, and easy operation [38, 39]. On the basis of different parameter responses, the piezoelectric quartz crystal sensors can be classified into two different types: quartz crystal microbalance and series piezoelectric quartz crystal [40–44]. The results acquired from the quartz crystal microbalance are usually by measuring the change in frequency of a quartz crystal resonator. The resonance is changed with the addition or removal of a small mass due to oxide growth/decay or film deposition at the surface of the acoustic resonator [45]. The quartz crystal microbalance can be used under vacuum, in gas phase and recently in liquid environments. It is very useful for monitoring the rate of deposition in thin film deposition systems under vacuum. In liquid, it is highly effective at determining the affinity of molecules (proteins, in particular) to surfaces functionalized as recognition sites. Larger entities such as *M. tuberculosis* and polymers are investigated, as well. Frequency measurements are easily made to high precision. Hence, it can be easy to respond to changes from mass loading on electrode surface down to a nanogram level [45]. Piezo-immunological sensor, which is recently developed on the basis of the quartz crystal microbalance technology, has been used for *M. tuberculosis* detection [46]. For example, He and zhang reported a novel piezo-immunological sensor for *M. tuberculosis* detection. In this method, the quartz crystal was first coated by the styrene-butadiene-styrene copolymer; the antibody was then successfully immobilized onto the membrane surface. After incubation with *M. tuberculosis*, the results were acquired based on the resonant frequency change. With this method, it can detect 10^5^ cells/mL *M. tuberculosis* [47]. Though this method is rapid, simple, and unlabeled, the results of this kind of sensors are easily affected by external factors, such as density, viscosity, dielectric constant, and conductivity of the solution [48]. The series piezoelectric crystal quartz sensor (SPQC), which is constructed by combining in series a pair of electrodes immersing in a liquid with a piezoelectric quartz crystal in oscillating circuit, is a very unique device in all of PQC sensors. It has a sensitive frequency response to electric parameters of the solution. SPQC sensor can respond to the changes of liquid conductivity with excellent frequency stability. Compared with conventional conductive methods, this method can detect a smaller conductivity change in the presence of electrolyte [49, 50]. Based on this superiority, the method has been extensively used as a highly sensitive biological and chemical sensor in various fields, such as the analysis of biochemical oxygen demand in environmental monitoring [38], the assay and detection of bacteria in food safety, clinic diagnosis, and so on [51]. He et al. also used it to detect and quantify *M. tuberculosis* H37Ra [39]. With the growth of *M. tuberculosis*, the conductivity of the culture medium was monitored using the sensor through the frequency response curve, where *x*-axis was the culture time and *y*-axis was the frequency shift. This method is rapid, sensitive, cheap, and the detection limit is as low as 2 × 10^3^ cells/mL. 

**Figure 1:**
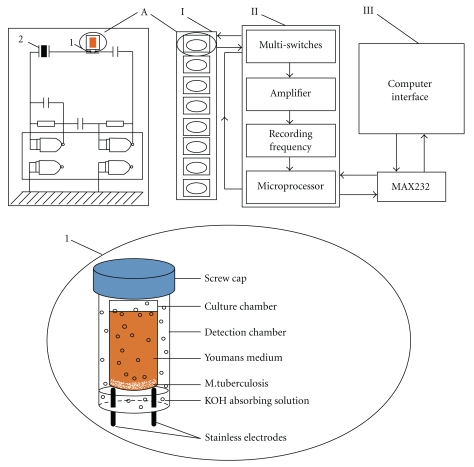
Block diagram drawing of the multichannel series piezoelectric quartz crystal sensor system. The system consists of 3 major components (I) eight samples detection system (A) the circuit of the single oscillator (1) detection cell; (2) 9 MHz AT-cut piezoelectric quartz crystal), (II) microprocessor system, (III) data output system; Reprinted with permission from [48]. Copyright 2008, Biosensors. Bioelectronics.

Recently, the potential use of volatile production patterns of *M. tuberculosis* and associated cells for early disease diagnosis including TB, urinary tract infections, and breast cancer based on electronic nose has been recognized [52–54]. Ren et al. has reported that the combination of the typical volatile production pattern produced by *M. tuberculosis* with the sensitive conductive response of the series piezoelectric quartz crystal sensor was a new automated continuous multichannel series piezoelectric quartz crystal (MSPQC) sensor system [48]. This system included a detection system for eight samples, a microprocessor system and data output system (Figure 1). In the detection system, it contained two chambers. One of the chambers was full of KOH absorbing solution with a pair of conductive electrodes at its bottom. The other chamber, which was inserted inside the detection chamber, was called the culture chamber with a special growth medium for *M. tuberculosis*. With the growth of *M. tuberculosis*, the component of the medium was decomposed into volatile NH_3_ and CO_2_. Then, the volatile NH_3_ and CO_2_ were absorbed by the KOH absorbing solution. The impedance changed in KOH solution was detected by the pair of conductive electrodes. It was connected to the piezoelectric quartz crystal coated with silver disc at two sides in series, which can change the oscillating frequency. This new automated, continuous and multichannel method was proposed for rapid separation and sensitive detection of *M. tuberculosis*. The detection limit was as low as 10 cells/mL. Compared with other methods, this system was more rapid, much cheaper and the detection limit is much lower. It can detect eight samples at the same time. Therefore, this method was promising to detect *M. tuberculosis* cheaply and quickly for clinic microbiological laboratories and other fields.

### 2.2. Electrochemical Biosensors

The field of electrochemical biosensors has significant growth in recent years due to their potential application for assay and detection of enzyme, nucleic acid, and microorganism [55, 56]. Usually in the detection process, the solid electrodes are used as the basic electrode. After the biological sensitive molecules are fixed on the electrode surface, the target molecules can be identified and captured onto the electrode surface through the specific recognition of biological molecules. The basic electrode, which works as a signal transmitter, can switch the signal produced by the specific recognition between biological molecules into the electrical signal including current, potential, impedance, and coulometry. Therefore, electrochemical biosensors can sensitively detect and quantify the analysis targets. The choice of the basis electrode is a crucial step for the development of biosensors. There is a wide variety of electrodes for choice to fabricate sensor devices, such as carbon paste electrodes [57], gold electrodes [58], and glassy carbon electrodes [59]. The immobilization of a biomolecule (e.g., DNA, and antibody) [60–63] onto a desired electrode surface is another important step. According to the immobilization of different biomolecules for *M. tuberculosis *detection, electrochemical biosensors can be divided into electrochemical immunosensors [61] and electrochemical DNA biosensors [60, 63]. 

Electrochemical immunosensors, which combine the high specificity of conventional immunochemical methods with electrochemical system, provides a potential opportunity to gain new insights to create sensitive and simple immunoassay devices for *M. tuberculosis* detection. Díaz-González et al. has developed an enzymatic voltammetric immunosensor for the determination of *M. tuberculosis* antigen [61]. A screen-printed carbon electrode, modified with the streptavidin, was used in this method as a signal transduction element. The biotinylated rabbit anti-*M. tuberculosis* antibodies were immobilized onto the electrode surface through the specific streptavidin-biotin reaction. In the presence of *M. tuberculosis* antigens and monoclonal antibodies against *M. tuberculosis*, a sandwich immune complexes of rabbit anti-*M. tuberculosis*/*M. tuberculosis* antigens/monoclonal antibodies against *M. tuberculosis* could be formed. The alkaline phosphatase (AP) labeled rabbit IgG anti-mouse immunoglobulin G, which was used as detector antibodies, was further adsorbed onto the monoclonal antibodies. When the 3-indoxyl phosphate was used as an electrochemical substrate, the resulting enzymatic product could cause the change of electrochemical behaviors on the electrode surface. Using this technique it was possible to detect *M. tuberculosis* antigens with detection limit of 1.0 ng/mL. 

**Figure 2:**
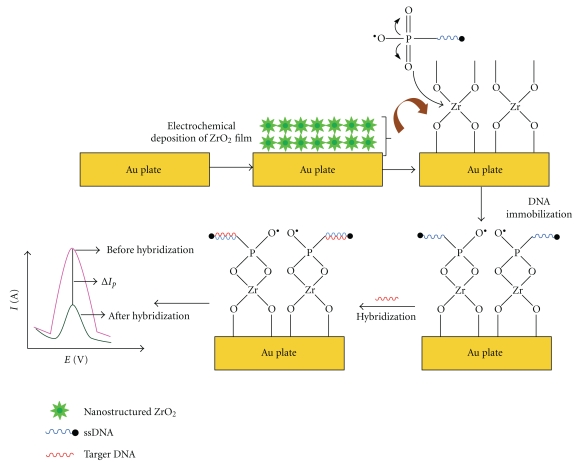
Proposed schematic for the fabrication of nano-ZrO_2_/Au-based DNA biosensor. Reprinted with permission from [63]. Copyright 2010, Applied Physics Letters.

Electrochemical DNA biosensors, which are based on nucleic acid hybridization, have attracted considerable attention due to their potential application for assay and diagnosis of TB and other diseases. Depending on the probes employed, the PNA (peptide nucleic acid) probes electrochemical biosensor [55] and DNA probe electrochemical biosensor have been developed [60, 63]. Nirmal Arora et al. has reported a method for *M. tuberculosis* detection using PNA probes [55]. In this method, the 21-mer PNA probe specific to 16 s–23 s rRNA spacer region of *M. tuberculosis*, has been covalently immobilized onto the polypyrrole-polyvinylsulphonate (PPy-PVS) film. The film was then electrochemically deposited onto indium-tin-oxide (ITO) glass to form the PPy-PVS/ITO electrode. The PNA probe was used for the hybridization detection with complementary sequence of *M. tuberculosis* DNA with a detection limit of 2.5 pg/*μ*L. The whole detection process can be finished within about 60 min. Das et al. detected *M. tuberculosis* using the Zirconia- (ZrO_2_-) based nucleic acid sensor [63]. ZrO_2_ is an attractive inorganic metal oxide with thermal stability, chemical inertness, nontoxicity, and affinity for groups which contain oxygen. These groups facilitate covalent immobilization without using any crosslinker which may limit the sensitivity of the fabricated sensor. So it is an ideal material to immobilize the biomolecules with oxygen groups [60, 62]. Moreover, ZrO_2_ has pH stability, which plays an important role as corrosion-resistant coatings on sensor applications. Based on the superiorities of ZrO_2_, the nanostructured ZrO_2_ film was electrochemically deposited onto the Au electrode surface to fabricate a DNA biosensor for *M. tuberculosis* detection. As can be seen in Figure 2, the ssDNA was the 21-mer oligonucleotide specific to *M. tuberculosis*. First, nano ZrO_2_ film was deposited onto the bare gold electrode [63, 64]. After the incubation of ssDNA with ZrO_2_/Au surface by utilizing the affinity between oxygen atom of phosphoric group and zirconium, the DNA biosensor was successfully fabricated. With this method, the detection limit was 0.065 ng/*μ*L and the whole detection process can be done within 60 s. Therefore, this kind of DNA-ZrO_2_/Au bioelectrodes can be used for early, sensitive and rapid assay, detection and diagnosis of *M. tuberculosis*.

### 2.3. Magnetoelastic Biosensors

The development and application of magnetoelastic biosensor techniques have been reported in the past few years [65–68]. Typically, this kind of sensors is a free-standing, ribbon-like magnetoelastic film coupled with a chemical or biochemical sensing layer such as enzyme. In response to an externally applied magnetic field, the sensor mechanically vibrates at a characteristic resonance frequency, launching a return magnetic field which can be remotely detected by a pickup coil. Since there is no physical contact between the sensor and the detection system, various magnetoelastic sensors have been developed for remote-query monitoring of different physical and chemical parameters (e.g., microorganisms [69], flow velocity [70], temperature [71], pressure [72], elasticity [73], mass loading [74–76], density, and liquid viscosity [77–79]). Pang et al. used the magnetoelastic biosensor for the direct, real-time detection and quantification of *M. tuberculosis *with a liquid medium. The sensor used in this method was fabricated by coating a magnetoelastic ribbon with a polyurethane protecting film [69]. With the growth and proliferation of *M. tuberculosis*, cells consumed the nutrients and decomposed the macromolecules into small molecules (e.g., CO_2_, NH_3_, organic acid and so on) within a liquid culture medium. Consequently, the medium properties, which were determined by the composition and concentration of the medium, could be changed by these small molecules. This, in turn, the resonant frequency of the magnetoelastic sensor was changed. Therefore, the sensor response sensitivity was affected by the characters of culture medium. Pang et al. discovered that the response at standard culture medium concentration was the best. On the basis of this concentration of the culture medium, they detected a linear relationship with the bacterial concentration in the range of 1 × 10^4^ to 1 × 10^9^ cells/mL. The detection limit was 10^4^ cells/mL. In addition, this method was also used in sputum sample. All of the results indicated that the magnetoelastic biosensor can be effectively used for monitoring and detecting the growth of *M. tuberculosis *both in culture medium and real samples because of its low cost and remote query nature.

## 3. Challenges and Perspectives

Analysis of published literatures has shown that the rapid development of biosensor technology has opened enormous opportunities for *M. tuberculosis* detection, as listed in Table 2. However, there are still many considerable challenges and issues remained for reliable and effective use in routine applications with biosensors. The biosensor system must have the adaptability and flexibility to detect different analytes with relatively simple and inexpensive structures. Although the sensitivity for detection of *M. tuberculosis *has been highly improved and the detection limit of *M. tuberculosis* can be as low as 10 cells/mL when detected by the new automated continuous multichannel series piezoelectric quartz crystal (MSPQC) sensor system [48], a biosensor must be able to provide a detection limit as low as single coliform organism in 100 mL of potable water [36]. Thus, the sensitivity is still a very important issue that requires improvement. Another problem is that the price of most of biosensors is too high to acquire, only a few biosensors for bacterial detection are relatively cheap to acquire and the main reasons for this are both the technology and market related reasons. This problem seriously influences the online detection of infectious bacteria (e.g., *M. tuberculosis*) worldwide. Especially for developing countries, this problem is more seriously. 

In recent years, with the successful development of nanotechnology, highly sensitive and accurate biosensor systems based on the combination of nanotechnology and biosensing technology have great application in the medical diagnostics, clinical medicine, environmental monitoring, food quality control, defense, and other industries. The applications of these biosensor systems have been also started to flourish in the field of *M. tuberculosis *detection. Therefore, the continued collaborations in various fields including chemistry, physics, materials science, molecular biology, and manufacture, will with no doubt speed up the translational process and eventually realize the great impact of biosensors for *M. tuberculosis* detection.

## Figures and Tables

**Table 1:** The various mentioned non-biosensing techniques for bacteria detection.

Method and technique type	Samples analyzed	Detection limit	References
PCR	*M. tuberculosis*	with the true positivity of 95.5%	Thomson et al. [7]
Latex agglutination	*M. tuberculosis*	with the true positivity of 73.6%	Krambovitis et al. [10]
ELISA	*M. tuberculosis*	with the true positivity of 68%	Delacourt et al. [13]
The AMTDT	*M. tuberculosis*	with the sensitivity of 94.3%	Gamboa et al. [15]
Radiometric detection	*M. tuberculosis*	—	Middlebrook et al. [14]
Flow cytometry	*M. tuberculosis*	3.5 × 10^3^ cells/mL	Qin et al. [18]
MB/Bact system	*M. tuberculosis*	—	Horvath et al. 2004
MB/Bact system	*Mycobacteria*	—	Cambau et al. [17]

**Table 2:** Different kinds of biosensors for *M. tuberculosis* detection.

Biosensor devices	Samples analyzed	Detection limit	References
Piezo-immunological sensor	*M. tuberculosis*	10^5^ cells/mL	He et al. [46]
The series piezoelectric crystal quartz sensor	*M. tuberculosis* H37Ra	2 × 10^3^ cells/mL	He et al. [39]
Multichannel series piezoelectric quartz crystal sensor	*M. tuberculosis*	10 cells/mL	Ren et al. [48]
Electrochemical immunosensor	*M. tuberculosis* antigens	1.0 ng/mL	Díaz-González et al. [61]
Electrochemical DNA biosensor	*M. tuberculosis* DNA	0.065 ng/*μ*L	Das et al. [63]
Magnetoelastic biosensor	*M. tuberculosis*	10^4^ cells/mL	Pang et al. [69]
Acoustic wave impedance biosensor	*M. tuberculosis*	2 × 10^3^ cells/mL	He et al. [39]
Surface plasmon resonance sensor	*M. tuberculosis* complex	30 ng/*μ*L	Duman et al. 2010
